# Dosimetric evaluation of unlaminated radiochromic films exposed to an Americium‐241 source using measurements and Monte Carlo simulations

**DOI:** 10.1002/mp.70001

**Published:** 2025-10-27

**Authors:** Mélodie Cyr, Maryam Rahbaran, Nada Tomic, Shirin A. Enger

**Affiliations:** ^1^ Medical Physics Unit Department of Oncology Faculty of Medicine McGill University Montreal QC Canada; ^2^ Research Institute of McGill University Health Centre Montreal QC Canada; ^3^ Lady Davis Institute for Medical Research Jewish General Hospital Montreal QC Canada

**Keywords:** alpha radiation, Americium‐241, film dosimetry

## Abstract

**Background:**

Radiochromic GafChromic film models are widely used in clinical settings for quality assurance during cancer treatment planning. Although these films are extensively studied in photon dosimetry, research on their application in α‐particle dosimetry remains limited. With the growing use of α‐particles in cancer therapy, it is important to establish film dosimetry protocols tailored to α‐particles. Unlike photons, α‐particles are charged, have a high linear energy transfer, and induce significantly greater biological damage, highlighting the need for specialized dosimetric approaches.

**Purpose:**

This study aimed to evaluate the response of various unlaminated GafChromic film models including EBT3, EBT‐XD, and HD‐V2, irradiated with an ^241^Am α‐particle source, with combined experimental film irradiation and Monte Carlo (MC) simulations.

**Methods:**

In this study, unlaminated EBT3, EBT‐XD, and HD‐V2 film pieces were irradiated with an ^241^Am disk source at various exposure times within a dark box. A detailed comparison was performed across the three film models, focusing on uncertainties and relative dose errors. Film analysis was conducted using a custom Python script, extracting normalized pixel values from the green channel. Additionally, a MC‐based user code was developed using the Geant4 simulation toolkit to model the ^241^Am source and calculate the dose rates in the active layers of the films and in water. The mean dose rates were also calculated in a 1 mm diameter region of interest. These simulated dose rates were employed to convert film exposure times into absorbed doses for both the active layers and water, establishing a reference dosimetry protocol for α‐particles across the three radiochromic GafChromic film models.

**Results:**

The mean dose rates within a 1 mm diameter circular region of interest in the active layers of the three unlaminated GafChromic film models were determined to be 3.77 ± 0.002 Gy/min for EBT3, 4.04 ± 0.0022 Gy/min for EBT‐XD, and 4.25 ± 0.0017 Gy/min for HD‐V2. When the film material was changed to water, the dose rate was increased 14.3% for EBT3, 19.2% for EBT‐XD, and 15.0% for HD‐V2, with EBT3 showing the closest match to water‐equivalence. Calibration curves for each film model were generated by fitting a power function to their responses. Refinements to the dose range were necessary to achieve an uncertainty below the 5% threshold. Among the models, HD‐V2 required the most adjustments to its dose range and exhibited the highest levels of experimental, fit, and total uncertainties, along with the largest relative dose errors.

**Conclusions:**

This study investigated α‐particle dosimetry protocols for unlaminated EBT3, EBT‐XD, and HD‐V2 GafChromic film models using experimental irradiations and MC simulations. Although EBT3 and EBT‐XD demonstrate strong potential for α‐particle quality assurance in treatment planning, the HD‐V2 film model requires further investigation before it can be recommended for this application.

## 1 INTRODUCTION

Radiochromic GafChromic films, introduced in the 1980s, provide accurate, high‐resolution radiation dose measurements. These films consist of a radiation‐sensitive monomer embedded in a water‐soluble polymer matrix coated between two polyester substrates. Upon radiation exposure, diacetylene molecules in the active layer undergo polymerization, forming polydiacetylene dye polymers, producing a blue color that absorbs light in the red and green spectrum. The degree of color change is a non‐linear relationship to the absorbed radiation dose and stabilizes within 24 h.^1^, ^2^, ^3^ Recent advancements in radiochromic GafChromic film models, such as EBT‐XD, incorporate larger crystals in the active layer to enhance light scattering, though this reduces spatial resolution.^2^

Ashland, Inc.^4^ has been the leading manufacturer of radiochromic GafChromic films, widely used in film dosimetry. Different models are optimized for specific dose ranges and energy conditions: HD‐V2 are designed for high‐dose applications (10–1000 Gy) for gamma and x‐ray radiation, and exhibit minimal energy dependence from 100 keV into the MV range; MD‐V3 film model is suited for moderate‐dose applications (1–100 Gy), with minimal energy dependence from 100 keV to the MV range; EBT3 and unlaminated EBT3 film models cater to lower‐dose measurements (0.2–10 Gy); EBT‐XD and unlaminated EBT‐XD films models are designed for extended‐dose applications (0.4–40 Gy); RTQA2 film models are primarily used for machine quality assurance (QA), this model covers a dose range of 0.2 to 10 Gy. All models perform effectively across 100 keV to MV energy ranges.^2^, ^4^ Unlaminated GafChromic films are intentionally produced without one protective polyester layer, exposing the active layer. This design is particularly suited for dosimetry involving radiation types that are significantly attenuated by the polyester substrate, such as α‐particles,^5^ ultraviolet photons,^6^ and protons with energies up to 10 MeV.^7^

The therapeutic potential of α‐particles for cancer treatment has recently gained renewed interest. α‐particle‐emitting radionuclides, such as ^223^Ra, ^224^Ra, ^225^Ac, ^212^Pb, ^227^Th, ^213^Bi, and ^211^Ac, are widely employed in targeted radiotherapy, where they are conjugated to chelates, antibodies, small molecules, or nanoparticles to deliver radiation precisely to cancer cells. This targeted approach deposits cytotoxic ionizing radiation within small cell clusters, single‐cell metastases, or small solid tumors, inducing DNA damage that ultimately leads to cell death, while sparing surrounding healthy tissue.^8^

QA is essential to radiation therapy across all modalities and radiation qualities, ensuring radiation safety, dosimetric accuracy, and effective treatment planning. However, the increasing use of α‐particles in radiation therapy presents unique physical and biological challenges that require specialized QA strategies to ensure optimal patient care and treatment efficacy. Radiochromic GafChromic films are frequently used for QA purposes.

GafChromic are widely recognized for their high spatial resolution and user‐friendly design, making them effective for quantifying photon and electron doses.^1^, ^9^, ^10^, ^11^ As α‐particles are increasingly utilized in cancer treatments, such as targeted alpha therapy and alpha‐emitting interstitial radiation therapy, there is a growing need to evaluate the response of GafChromic film to α‐particles. However, the unique characteristics of α‐particles, including their short range in matter and high linear energy transfer (LET), present challenges for film‐based dosimetry. Despite the potential applications, the dosimetric response of GafChromic films to α‐particle irradiation remains poorly characterized.

To date, limited studies have explored α‐particle film dosimetry. Research includes one study on HD‐V2 films,^9^ a single study on laminated and unlaminated EBT2 films,^10^ five on unlaminated EBT3 films^11^, ^12^, ^13^, ^14^, ^15^ and one study on RT‐QA2 and XR‐QA2 films.^16^ Among these, unlaminated EBT3 films were the most extensively studied, often employing the peeled‐off method to unlaminate the standard EBT3 film. However, there is a notable gap in the literature where a direct comparison of the dynamic dose ranges for different film models under α‐particle irradiation has not been established. Moreover, recent findings suggest that the quenching effect—a reduction in film sensitivity due to high‐LET particles—significantly influences the response of GafChromic films to alpha particles. These findings underscore the need to develop dedicated α‐particle dosimetry protocols to characterize film responses accurately.

The first study exploring this application was conducted by Aydarous et al. (2013)^9^ with the irradiation of HD‐V2 films with an ^241^Am plane source. This initial work was followed by several subsequent studies with various film models, further expanding on the topic.^5^, ^10^, ^11^, ^12^, ^13^, ^14^, ^15^, ^16^ More recently, Diaz‐Martinez et al. (2024)^5^ investigated the response of unlaminated EBT3 GafChromic film model irradiated with an ^241^Am disk source and compared it to 6 MV photons. The authors used Monte Carlo (MC) simulations to establish the dose rates in the film, generating calibration curves for experimental α‐particle film dosimetry and reported an under‐response of unlaminated EBT3 GafChromic films to α‐particle irradiation when compared to their response to 6 MV photon irradiation. This phenomenon, is characterized as the “quenching effect” which occurs because high‐LET particles such as α‐particles, deposit most or all of their energy within a single polymerization site, limiting the propagation of polymerization events that typically occur with photon irradiation.

The LET dependence of GafChromic films was further demonstrated by Anderson et al. (2019),^17^ who irradiated EBT3 films using a proton therapy beam at varying energies. Their study revealed that the dosimetric response of the films decreased with increasing LET, with an under‐response of ∼15%, a clinically significant discrepancy. They recommended applying corrector factors to account for this effect when using GafChromic films for proton therapy dosimetry to ensure accuracy.^17^ In another study, Lee et al. (2018)^15^ employed MC simulations to model α‐particle exposure in a cell culture dish irradiated with an ^241^Am disk source, validating their simulations with experimental EBT3 film exposures and establishing dose‐distribution calibration curves.

This study aims to address this gap by systematically investigating the dosimetric response of several unlaminated GafChromic film models, including EBT3, EBT‐XD, and HD‐V2, irradiated by an ^241^Am α‐particle disk source. The dose rate for each film was calculated using MC simulations, and the corresponding dose profiles were generated based on the simulated dose rates.

## 2 METHODS

### 2.1 Unlaminated GafChromic film models

For this study, unlaminated EBT3 (Ln 06171901), EBT‐XD (Ln 11221902P1), and HD‐V2 (Ln 01091801) GafChromic film models were selected due to their widespread application in clinical dosimetry QA. The unlaminated EBT3 and EBT‐XD film models were customordered from Ashland.^4^ The HD‐V2, provided unlaminated, was also ordered from Ashland. These unlaminated models of GafChromic films feature an active layer on a single protective polyester backing, lacking the additional protective polyester layer present in standard GafChromic films. This configuration allows the emitted α‐particles from the ^241^Am source, with an average energy of 5.46 MeV and a limited range in the water of roughly 40–60 µm, to reach the active layer directly. Table 2 lists the specific compositions of the unlaminated EBT3, EBT‐XD, and HD‐V2 models.

### 2.2 Americium source composition

**FIGURE 1:**
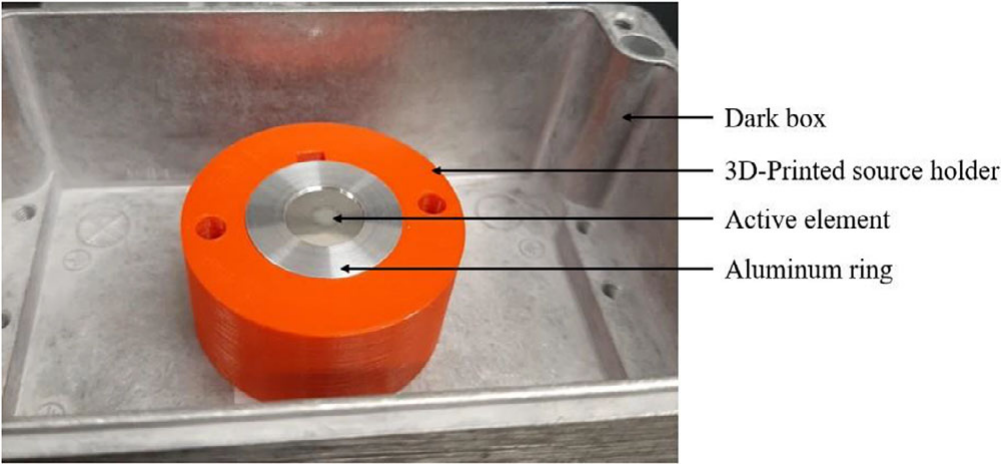
Photograph of the ^241^Am disk source used for film irradiation. The source sits in a box to shield the setup from ambient light, effectively simulating a black box environment, preventing unintentional activation of the light‐sensitive film during long irradiation times. The disk source sits in a 3D‐printed holder (orange) to avoid direct manipulation of the disk.

An ^241^Am type A‐1 disk with a current activity of 48 kBq (half‐life = 432.2y) purchased from Stuart Hunt & Associates Ltd. (Mississauga, Ontario, Canada) was used in this study. The active ^241^Am is permanently fixed in an aluminum holder 25.4 mm diameter × 3.18 mm high. The active element is embedded in platinum and positioned inside the aluminum holder. The active area is a circle of 5.0 mm in diameter and 1.08 nm in thickness. A 3‐D printed holder was fabricated to hold the disk source in place during film irradiation, with a lid to protect the source when it is not in use. Figure 1 shows a photograph of the source.

### 2.3 Monte Carlo simulations

The developed MC software was based on Geant4 10.02.p02^18^ and utilizes multi‐threading to increase the speed of simulations. The radioactive decay module of Geant4 was used to directly sample the decay scheme of ^241^Am. The decay products after ^237^Np were excluded from the simulations as ^237^Np is a stable long‐lived radionuclide with a half‐life of 2.14 million years, and therefore its decay products are not seen experimentally.^19^ Simulations were performed on the Cedar supercomputing cluster of the Digital Research Alliance of Canada. The dose was scored in the active layer of each film in 0.01 × 0.01 × 0.014 mm^3^ for the EBT3 film and 0.01 × 0.01 × 0.012 mm^3^ voxels for the EBT‐XD and HD‐V2 films. A summary of the MC simulation parameters is presented in Table 1 per the recommendations of the AAPM 268 report.^20^ The film elemental compositions used in the MC simulations are shown in Table 2.

**TABLE 1:** The Monte Carlo parameters used in this study.

Item	Description	Reference
**Toolkit**	Geant4 10.02.p02	^18^
**Validation**	Comparison to Diaz–Martinez *et al.*	^5^
**Timing**	4 h per batch (total 250 batches)	
**Source description**	^241^Am type A‐1 disk (Stuart Hunt & Associates Ltd)	
**Cross‐sections**	EPDL97, EEDL97, EADL97	^21^, ^22^, ^23^
**Transport parameters**	G4EmStandardPhysics option4 electromagnetic physics list, with 0.001 mm cut‐offs for photons.	^24^
**Variance reduction techniques**	None	
**Scored quantities**	Absorbed dose per decay in film material and water.	
**Number of histories and statistical uncertainties**	2 × 10^8^ histories per batch (1010 total), < 1% average uncertainty per voxel in a 1 mm diameter circular region of interest	
**Statistical methods**	History‐by‐history	^25^
**Post‐processing**	Normalization of dose rate to Gy/min for a 48 kBq source	

**TABLE 2:** Elemental compositions for the unlaminated EBT3, EBT‐XD, and HD‐V2 films.

		Active layer			Polyester layer		
		EBT3	EBT‐XD	HD‐V2	EBT3	EBT‐XD	HD‐V2
Thickness (mm)		0.014	0.012	0.012	0.125	0.125	0.097
**Element (%)**	**H**	8.65	8.83	10.80	4.20		
	**Li**	0.63	0.65	1.80	–		
	**C**	50.01	52.98	79.00	62.50		
	**N**	0.64	0.86	–	–		
	**O**	32.37	29.00	8.4	33.30		
	**Na**	0.35	0.36	–	–		
	**Al**	6.57	6.27	–	–		
	**S**	0.24	0.49	–	–		
	**Cl**	0.54	0.55	–	–		
**Density (g** *·* **cm** *^−^* ^3^ **)**		1.15	1.35	0.95	1.35		

### 2.4 Film irradiation and scanning

The unlaminated EBT3, unlaminated EBT‐XD, and unlaminated HD‐V2 GafChromic film models were irradiated using the previously described ^241^Am disk source. Films were prepared by cutting them into 5.08 × 6.35 cm pieces using a guillotine paper cutter. A consistent landscape orientation was maintained throughout the study: the films were cut along their longitudinal side to a length of 6.35 cm, then rotated and cut to a width of 5.08 cm, ensuring a clear distinction between their dimensions.

**FIGURE 2:**
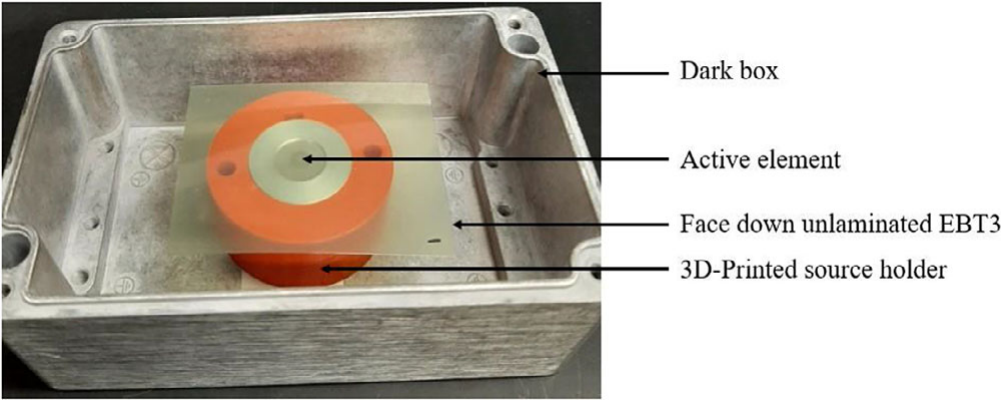
Film irradiation experimental set‐up. The piece of unlaminated EBT3 film sits face down directly on top of the ^241^Am source, in the dark gray box.

Before irradiation, the films were scanned using an Epson Expression 11000XL flatbed document scanner at 127 dpi in 48‐bit RGB (Red‐Green‐Blue) mode (16‐bits per color) and saved as Tagged Image File Format (.tiff). During irradiation, the films were positioned with the active layer facing down, directly over the ^241^Am source, which was housed inside a dark gray box, as illustrated in Figure 2. The box remained closed during exposure to prevent any unintentional UV light interference.

Irradiation times (*t*) varied across film types, corresponding to the dose ($D_{MC}$) in Gy, which was calculated by multiplying the dose rates (${\dot{D}}_{MC}$) determined through MC simulations, defined by the Equation 3. The films were irradiated in triplicate to reduce the experimental uncertainty

(1) $$\;D_{MC}=t\cdot {\dot{D}}_{MC}$$

The scanned film images were analyzed using a custom Python 3.8.3 script. A region of interest measuring 1 × 1 mm^2^ (5 × 5 pixels) was used to extract pixel values (PV) from the green color channel of each film image. Following a methodology similar to that of Diaz–Martinez et al. (2024),^5^ normalized pixel values ($PV_{norm}$) were calculated for each film using the Equation 2.

(2) $$P\;V_{norm}=\left(\frac{PV_{background}}{PV_{irradiated}}-1\right)\;$$

Where, $PV_{background}$ are the pixel values from the films prior to irradiation and $PV_{irradiated}$ are the pixel values from the irradiated films.

The $PV_{norm}$ were then plotted as a function of dose (Gy), and calibration curves were generated by fitting the data using the Equation 3. The film responses were subsequently compared by plotting the dose calculated by both the dose rates in the active layer of the films against the corresponding dose rates in water. This fitting approach, originally developed for photon irradiation by Devic et al. (2004),^26^ has been recently validated for which was used in α‐particle dosimetry by Diaz–Martinez et al. (2024).^5^

(3) $$D=a\left(PV_{norm}\right)+b{\left(PV_{norm}\right)}^{n}$$

Where D is the dose in Gy, $PV_{norm}$ are the normalized pixel values, and *a*, *b*, *n* the fitting parameters of the power function, where a was always forced to be zero.

The error analysis followed the approach outlined by Devic et al. (2004).^26^ Dose uncertainties were calculated by combining the relative fit uncertainties of the fit parameters with the experimental uncertainties, which were added in quadrature. Additionally, a comprehensive uncertainty and error analysis was conducted, plotting the experimental, fit, and total uncertainties alongside the relative dose error. This method was used to identify potential outliers in the dosimetric protocol. The error analysis is presented below:

The relative experimental uncertainty of the measured dose for the established function form given by Equation 3 is given by

(4) $$\sigma_{D_{exp}}\;\left(\%\right)=\frac{\sqrt{{\left(a+n\cdot b\cdot PV_{norm}^{n-1}\right)}^{2}\cdot \sigma_{PV_{norm}}^{2}}}{D_{fit}}\;\times 100$$

where $D_{fit}$ is the dose acquired from the fit function 3, using the acquired parameters from the calibration curves. The Equation 5 is relative fit uncertainty expressed as

(5) $$\sigma_{D_{fit}}\;\left(\%\right)=\frac{\sqrt{{\left(PV_{norm}\right)}^{2}\cdot \sigma_{a}^{2}+{\left(PN_{norm}\right)}^{2\cdot n}\cdot \sigma_{b}^{2}}}{D_{fit}}\;\times 100$$

There is an added uncertainty when converting the irradiation time into dose using the dose rates calculated in the MC simulations. This relative MC uncertainty is calculated as

(6) $$\sigma_{D_{MC}}\;\left(\%\right)=\frac{\sqrt{\left(\sigma_{MC}^{2}\cdot t^{2}\right)+\left(\sigma_{t}^{2}\cdot {\dot{D}}_{MC}^{2}\right)}\;}{D_{fit}}\;\times 100$$

The total relative uncertainty for the dose measured using the above‐described formalism for the functional form given by Equation 3 is calculated as

(7) $$\sigma_{D_{tot}}\;\left(\%\right)=\frac{\sqrt{{\left(a+n\cdot b\cdot PV_{norm}^{n-1}\right)}^{2}\cdot \sigma_{PV_{norm}}^{2}+{\left(PV_{norm}\right)}^{2}\cdot \sigma_{a}^{2}+{\left(PN_{norm}\right)}^{2\cdot n}\cdot \sigma_{b}^{2}+\left(\sigma_{MC}^{2}\cdot t^{2}\right)+\left(\sigma_{t}^{2}\cdot {\dot{D}}_{MC}^{2}\right)\;}}{D_{fit}}\;\times 100$$

The relative dose error between the experimental dose (*D*) and theoretical dose ($D_{fit}$) is determined as

(8) $$\delta_{Error}\;\left(\%\right)=\left|\frac{D_{fit}-D}{D_{fit}}\right|\;\times 100$$

## 3 RESULTS

### 3.1 Monte Carlo simulations

**FIGURE 3:**
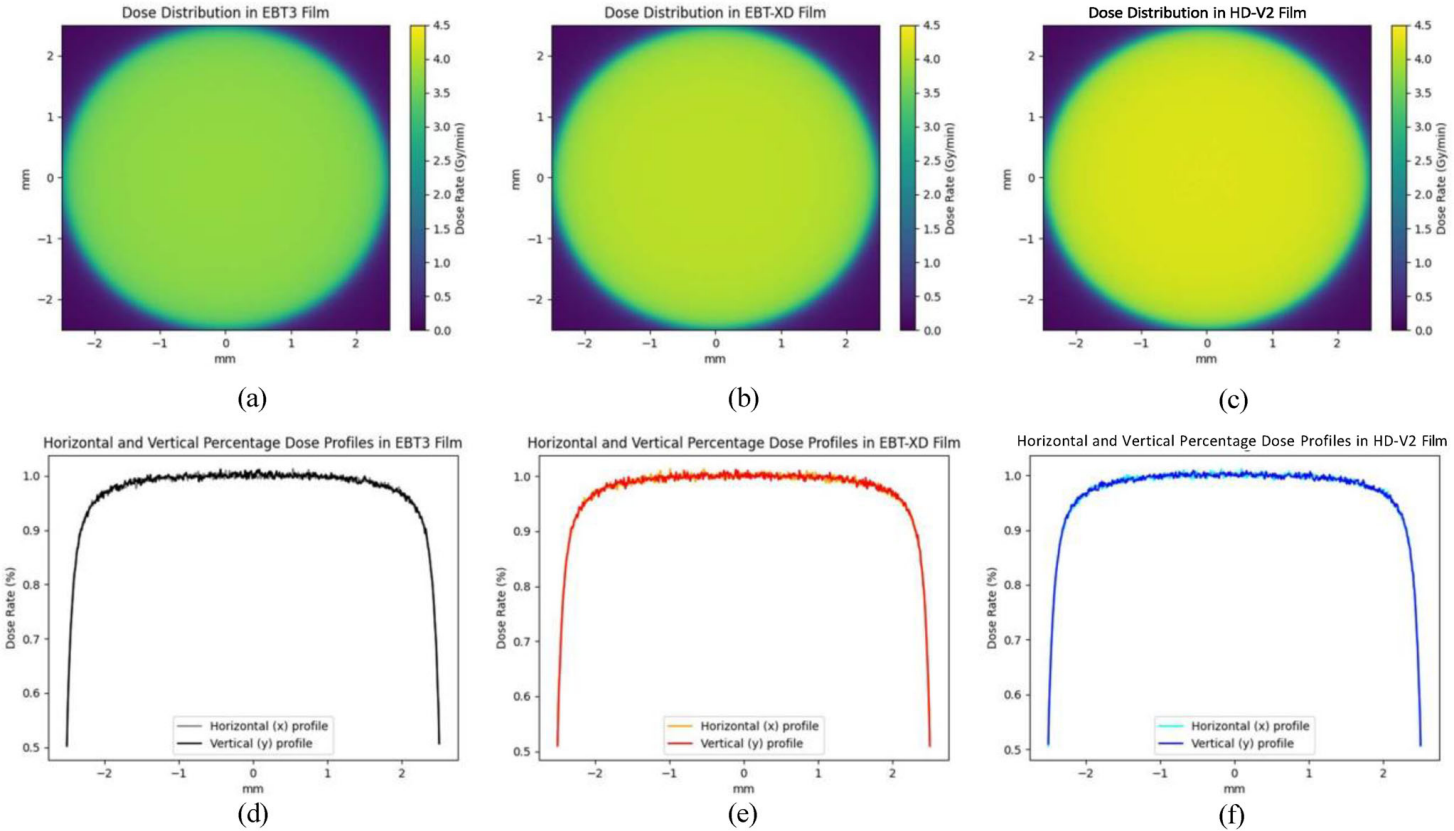
Dose distributions calculated with the Monte Carlo method in film‐specific materials for unlaminated EBT3, EBT‐XD, and HD‐V2 films (a–c) and horizontal and vertical line dose profiles, normalized to the mean dose rate in a 1 mm circular ROI in the center of the films (d–f).

The mean dose rates calculated in a 1 mm circular region of interest in film materials and water are presented in Table 3 for unlaminated EBT3, EBT‐XD, and HD‐V2 films. Figure 3 shows the dose distributions in the active layers of the respective films.

**TABLE 3:** Summary of mean dose rate results for the EBT3, EBT‐XD, and HD‐V2 films on the surface of the source in a 1 mm circular ROI.

Simulation		Dose rate (Gy/min)	Statistical uncertainty (%)
**EBT3**	Film	3.77	0.20
	Water	4.40	0.40
**EBT‐XD**	Film	4.04	0.22
	Water	5.00	0.40
**HD‐V2**	Film	4.25	0.17
	Water	5.00	0.44

### 3.2 Film response

**FIGURE 4:**
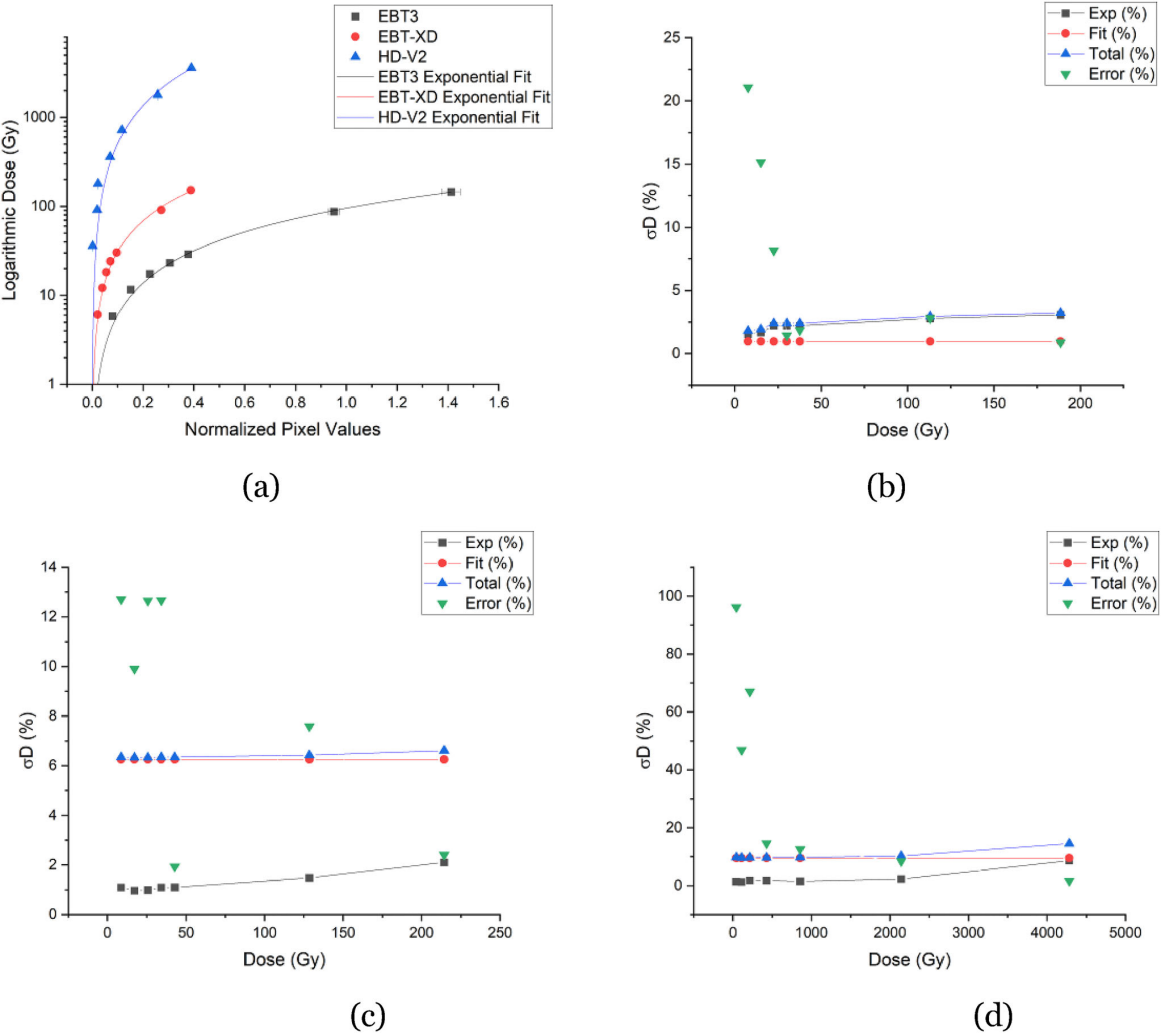
Calibration curves obtained using the absorbed dose to water for all film responses (a). The absorbed dose to water (Gy), represented in logarithmic scale, is plotted as a function of normalized pixel values. Uncertainty analyses for the respective film models– EBT3 (b), EBT‐XD (c), and HD‐V2 (d)–are presented. These analyses detail the experiment uncertainty (Exp), fit uncertainty (Fit), total uncertainty (Total), and relative dose error (Error), all expressed as percentages and relative to dose (Gy).

Each film exhibited a distinct response to the irradiation and covered different dose ranges. The calibration curves for each film, reflecting the active layer dose rates derived from MC simulations, closely followed the response calculated in water. Given that clinical recommendations prioritize dose in water, the water‐based dose film responses were reported based on water‐equivalent conditions. In Figure 4, the data points were fitted with a power function, with corresponding error bars included; however, some error bars may be too small to discern.

**FIGURE 5:**
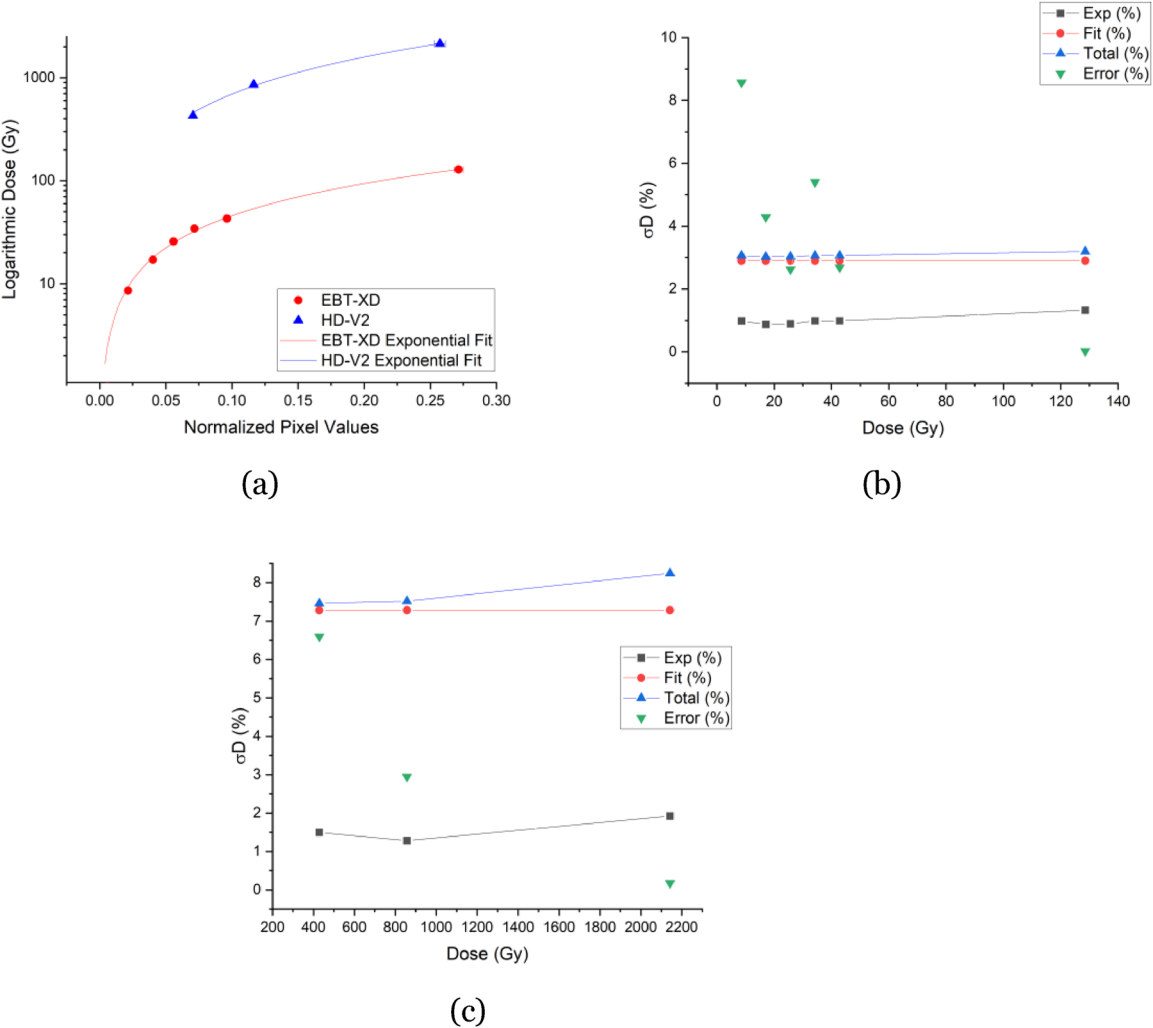
Calibration curves obtained using the dose simulated for water for the refined dose range of EBT‐XD and HD‐V2 film models (a). The dose (Gy) is plotted on a logarithmic scale as a function of normalized pixel values. The uncertainty analyses for the respective film models–EBT‐XD (b) and HD‐V2 (c)–are presented. These analyses detail the experiment uncertainty (Exp), fit uncertainty (Fit), total uncertainty (Total), and relative dose error (Error), all expressed as percentages and relative to dose (Gy). Refined film responses with water dose rates with uncertainties.

Upon further investigation, it was observed that the fit uncertainties were higher than the 5% threshold^1^, ^2^ for the EBT‐XD and HD‐V2 films, prompting adjustments to their calibration curves. The EBT3 film calibration curve, however, remained unchanged. By removing the highest dose of 173.2 ± 0.38 Gy from EBT‐XD, as well as removing doses from both low and high values from HD‐V2 (resulting in a dose range of 364.2 ± 0.62 Gy to 1821.2 ± 3.10 Gy), the fits for EBT‐XD and HD‐V2 film models improved, resulting in lower $\chi^{2}$ values and reduced fit uncertainties. Hereon, this reduction in data points to improve the fit for the EBT‐XD and HD‐V2 film models, will be referred to as refining/refined dose ranges. Updated figures were generated for the film response to the dose, calculated from the water‐based dose rate, presented in Figure 5. The data points were fitted with a power function, with corresponding error bars included; however, some error bars may be too small to discern. The reduction in data points for the HD‐V2 film highlights the need for additional experiments to enhance the data density along the fit. The parameters derived from the calibration curves for EBT3, as well as the refined EBT‐XD and HD‐V2 films, are summarized in Table 4. The fit parameters reflect the refined EBT‐XD and HD‐V2 data to improve accuracy and reduce uncertainties.

**TABLE 4:** Parameters derived from the power fit function for each film type and simulated dose rate composition (active layer of the films or in water). Included in the table is the parameter b with its associated fitting error ($\sigma_{b}$), the parameter *n* with its corresponding fitting error ($\sigma_{n}$), and the reduced chi‐squared value ($\chi^{2}$), providing a measured of the goodness of fit.

Film type		*b*	*σ*_b_	*n*	*σ*_n_	*χ*2
**EBT3**	Film	105.8	1.02	1.20	0.02	3.0
	Water	123.5	1.19	1.20	0.02	4.09
**EBT‐XD**	Film	401.0	11.6	1.04	0.02	1.20
	Water	496.5	14.4	1.04	0.02	1.81
**HD‐V2**	Film	9245.6	673.0	1.20	0.05	1049.4
	Water	10877.1	791.8	1.20	0.05	1452.5

The calibration curve uncertainties improved after refining the EBT‐XD and HD‐V2 films, although the HD‐V2 film uncertainties remained relatively high, with a baseline of 8% for both fit and total uncertainties. The relative dose error was reduced to 7%, as shown in Figure 5c, representing a significant improvement from the initial baseline of 9.6% and a relative dose error as high as 96% (Figure 4d). Despite these improvements, a 7% error remains considerable, as the acceptable threshold for photon film dosimetry is typically 5%.^1^, ^2^ These findings suggest that the HD‐V2 film, when irradiated with an ^241^Am source, is not well‐suited for distinguishing low doses with high accuracy. Additionally, the $\chi^{2}$ values for HD‐V2 films were significantly higher than the EBT3 and EBT‐XD, as summarized in Table 4. The optimal dose range for each film type is listed in Table 5, where the HD‐V2 films was determined to be 364.2 ± 0.62 to 1821.2 ± 3.10 Gy, based on the refined calibration curve.

**TABLE 5:** Optimal dose range (in Gy) for the three film types, when irradiated with an α‐emitting ^241^Am disk source, determined using the film responses.

Film type	Minimal dose (Gy)	Maximal dose (Gy)
**EBT3**	22.6 ± 0.09	188.6 ± 0.75
**EBT‐XD**	17.08 ± 0.08	128.6 ± 0.57
**HD‐V2**	364.2 ± 0.62	1821 ± 3.10

Experimental uncertainties remained low for the EBT‐XD film model, similar to those observed with the HD‐V2 and EBT3 film models. However, the fit and total uncertainties increased to approximately 3% (Figure 5b), which is within the acceptable threshold of 5%. After refinement, excluding the highest dose value, the uncertainties were further reduced to reasonable levels. When the higher dose value was included, the fit and total uncertainties exceeded 6% (Figure 4d), which is considered high. Additionally, relative dose errors were as high as 13% but decreased to 9% following refinement. Notably, a significant error persisted at the dose value of 8.6 ± 0.04 Gy. Therefore, the optimal dose range for the EBT‐XD film was identified as 17.08 ± 0.08 to 128.6 ± 0.57 Gy (Table 5).

The EBT3 film model demonstrated experimental, fit, and total uncertainties (Figure 4b) all under the acceptable 5% uncertainty threshold. However, the relative dose error was initially high, with a 21% error observed at 7.6 ± 0.03 Gy and 15% at 15.0 ± 0.06 Gy. Within the optimal dose range of 22.6 ± 0.09 to 188.6 ± 0.75 Gy (Table 5), the relative dose error decreased significantly, ranging from 8% to 0.9%.

## 4 DISCUSSION

The increasing clinical application of α‐particles has highlighted the need to develop robust α‐particle dosimetry protocols to characterize better the response of unlaminated radiochromic GafChromic film models to α‐particle radiation. Although dosimetry protocols for radiochromic GafChromic films are well‐established in photon external beam radiation therapy,^1^, ^26^, ^27^, ^28^ Diaz‐Martinez (2024)^5^ demonstrated that calibration curves derived from photon film dosimetry can also be adapted for α‐particle dosimetry. Building on this foundation, the present study investigates the response of three unlaminated radiochromic GafChromic film models—EBT3, EBT‐XD, and HD‐V2—for α‐particle dosimetry. Their performance is evaluated against literature and using a newly designed ^241^Am disk source.

### 4.1 Monte Carlo simulations

The simulated dose rate was highest in the active layer of the HD‐V2 film. The simulated dose rate was homogeneous in the horizontal and vertical directions across the source diameter, with a steep fall‐off at the edge of the source, as shown in Figure 3. The percentage difference between the dose rate in water and in film‐specific materials was 14.3%, 19.2%, and 15.0% for the EBT3, EBT‐XD, and HD‐V2 films, respectively. Among the three, the EBT3 film exhibits the closest water‐equivalence, while the EBT‐XD films demonstrate the largest deviation, rendering it the least water‐equivalent for α‐particle film dosimetry. This is likely due to the higher atomic number elements present in the active layer crystals, such as sodium aluminum in the EBT‐XD and EBT3 films, attenuating more dose compared to water. In addition, all of the films have a high mass percentage of carbon present in the active layer crystals, which has a higher stopping power than water in the energy range of the α‐particles emitted by ^241^Am and therefore could be a cause of the significant difference between doses to film and water.^29^ The dose deposited in the EBT3 and HDV2 film active layers were the lowest and highest, respectively. Notably, the elemental mass composition of carbon in the EBT3 and HD‐V2 films was the lowest and highest, respectively. Therefore, one of the causes of the difference in the dose deposited in a film active layer material could be attributed to the elemental mass composition of carbon.

A study by Srinivasan et al. (2019)^30^ noted a significant difference between doses of film and water for EBT and EBT2 radiochromic GafChromic films with electrons, for energies lower than 100 keV.^30^ However, this effect was observed for lower energy electrons, and our study focuses on higher energy α‐particles. In addition, Martisikova et al. (2010)^31^ studied the water‐equivalence of EBT3 radiochromic GafChromic films with heavy carbon ion therapeutic beams and found that the water‐equivalence is energy‐dependent.^31^ It is, thus, possible that the water‐equivalence of radiochromic GafChromic films is energy‐dependent for α‐particles as well. Therefore, further investigation of water‐equivalent film material for α‐particle dosimetry is necessary.

### 4.2 Experimental film response

According to Ashland,^4^ the GafChromic films used in this study are optimized for specific photon dose ranges: laminated EBT3 for 0.2–10 Gy, laminated EBT‐XD films for 0.4‐40 Gy, and HD‐V2 films for doses up to 1000 Gy. However, their dose response under α‐particle irradiation, especially for unlaminated variants, remains less explored.

In this study, we observed increased relative dose errors at lower doses across all film types, often exceeding the 5% acceptable uncertainty threshold. At the lower doses, the polymerization‐induced color changes in the films result in pixel values that are similar to those of the non‐irradiated background. Consequently, when calculating the difference between the signal and background, the results often fall within the uncertainties, making it challenging to distinguish the radiation response from noise. Additionally, the flatbed scanner used in this study, which operates at a standard resolution of 127 dpi, further limits the sensitivity to lower doses. Such flatbed document scanners, typically designed for photocopying and office use, introduce additional artifacts, such as light scattering, that can distort measurements and exacerbate errors at low doses. These findings, similarly found in other studies,^1^, ^5^, ^32^ emphasize the need for a lower dose restriction when using radiochromic GafChromic films for α‐particle dosimetry. Furthermore, HD‐V2 film models, in particular, exhibited the highest relative dose errors. Although the active component in HD‐V2 is chemically the same as that in EBT3 and EBT‐XD, the crystalline structure differs, resulting in an absorbance peak near 670 nm rather than 635 nm for EBT3 and EBT‐XD. In the HD‐V2 active layer, the polymer matrix exhibits structural deformations when irradiated, such as chain aggregation, and is less amorphous than its counterparts.^32^, ^33^ These differences in the polymer matrix contribute significantly to higher uncertainties, relative dose errors, and discrepancies in the film's dosimetric response.

Diaz‐Martinez (2024) et al.^5^ investigated unlaminated GafChromic EBT3 films with an ^241^Am source. Their source featured a gold cover over the active element, had a different activity level, but emitted α‐particles with the same average energy. They identified a dose range of 17.0 to 91.0 Gy. In contrast, our study, using a higher‐activity, uncovered ^241^Am source, found a broader range of 22.6 to 188.6 Gy, using the MC simulated dose rates in water. A key difference lies in the dose ranges investigated; Diaz–Martinez did not explore doses between 91 and 549 Gy, allowing our study to identify a broader range. Additionally, our ^241^Am source lacked the gold cover, which will attenuate α‐particles. These distinctions contributed to the differences in the observed dose ranges and relative dose errors.

For unlaminated EBT‐XD GafChromic film model, our study identified an optimal dose range of 17.1–128.6 Gy, based on MC‐simulated dose rates in water. Although this overlaps with the EBT3 response, EBT‐XD films displayed more erratic in uncertainties and relative dose errors. To our knowledge, this is the first study evaluating unlaminated EBT‐XD under α‐particle irradiation. EBT‐XD film models, introduced in 2015, share a similar composition with EBT3 films but feature smaller crystals in their active layer, reducing their sensitivity, making EBT‐XD films more suitable for high‐dose measurements.^2^, ^34^ The smaller crystals in the active layer may play a role in the increased variability in polymerization events, particularly when exposed to α‐particles. Due to their distinct path lengths, α‐particles may not consistently interact with multiple crystals, resulting in less uniform responses. Additionally, this behavior is compounded by the quenching effect,^5^, ^17^ where the high LET of α‐particles leads to an under‐response of the films, further contributing to variability and reduced sensitivity.

For the unlaminated HD‐V2 radiochromic GafChromic film model, our study identified an optimal dose range of 364.2 to 1821.2 Gy, based on MC‐simulated dose rates in water. A similar study by Aydarous et al. (2013)^9^ examined the HD‐V2 radiochromic GafChromic film model using an ^241^Am source with an average α‐particle energy of 5.5 MeV. Their findings indicated saturation of the green channel at doses exceeding 1000 Gy, recommending using the red channel for doses up to 330 Gy.

In our study, we did not analyze the red channel but observed that the green channel produces measurable results at 364.2 ± 0.62 Gy, consistent with the suggestion that the red channel may be more suitable for lower doses. The differences in the dose ranges between the two studies can be attributed to variations in the physical properties of the HD‐V2 films used. The films in our study consisted of a back polyester based with an exposed active layer of 12 µm thickness and a density of 0.95 g/cm^3^. In contrast, the films used by Aydarous et al. (2013),^9^ had an active layer of 8 µm thick, a density of 1.2 g/cm^3^, and a clear 97 µm polyester substrate as a protective backing. The increased thickness and lower density of the active layer in our films likely contributed to the observed higher dose range. These differences suggest a greater capacity for polymerization events in our films due to the increased volume of the active layer, potentially enhancing their response to higher doses.

## 5 CONCLUSIONS

This study investigated α‐particle dosimetry protocols for unlaminated EBT3, EBT‐XD, and HD‐V2 radiochromic GafChromic film models using a combination of experimental irradiations and MC simulations. Dose rates derived from MC simulations varied between the active layers of the films and water for each model. These dose rates were used to convert irradiation times into corresponding doses, enabling comparisons of water‐equivalent dose ranges across the different film models. The findings demonstrated that EBT3 exhibited the closest dose rate agreement with water and the highest sensitivity, while EBT‐XD film model showed the greatest disparity in dose rate from water and an intermediate film response. The HD‐V2 supported the highest doses and showed higher uncertainties and relative dose errors compared to EBT3 and EBT‐XD. These results highlight the need for further experiments to improve the calibration curve fitting model for HD‐V2 films.

## CONFLICT OF INTEREST STATEMENT

The authors have no conflicts to disclose.
